# The hyphae-specific C_2_H_2_ transcription factor HscA regulates development, stress response, and mycotoxin production in *Aspergillus* species

**DOI:** 10.1128/msphere.00254-25

**Published:** 2025-06-10

**Authors:** Ye-Eun Son, Kyu-Hyun Kim, He-Jin Cho, Jae-Hyuk Yu, Hee-Soo Park

**Affiliations:** 1 School of Food Science and Biotechnology, Kyungpook National University 34986, Daegu, South Korea; 2 Department of Bacteriology, University of Wisconsin 205263, Madison, Wisconsin, USA; 3 Department of Integrative Biology, Kyungpook National University 34986, Daegu, South Korea; 4 Department of Advanced Bioconvergence, Kyungpook National University 34986, Daegu, South Korea; University of Georgia, Athens, Georgia, USA

**Keywords:** *Aspergillus nidulans*, *Aspergillus flavus*, transcription factor, Cys_2_His_2_, zinc finger domain

## Abstract

The zinc cluster family is the largest group of transcription factors involved in regulating fungal growth, morphology, and differentiation in *Aspergillus* species. In this study, we investigated hyphae-specific zinc cluster transcription factors and characterized a novel hyphae-specific Cys_2_His_2_ zinc finger transcription factor, designated HscA, in the model fungus *Aspergillus nidulans* and the toxigenic fungus *Aspergillus flavus*. Phenotypic analyses demonstrated that HscA is essential for normal asexual and sexual development in *A. nidulans* and *A. flavus*. Deletion of *hscA* resulted in elevated sensitivity to cell wall stress agents and an ion depletion stressor. Moreover, the *hscA* null mutant exhibited decreased production of sterigmatocystin in *A. nidulans* and aflatoxin B1 in *A. flavus*. Conidial production in the kernel was decreased in the Δ*hscA* strain compared to the control in *A. flavus*. Overall, these results suggest that HscA plays a pivotal role in fungal development, stress tolerance, and mycotoxin production in *Aspergillus* species.

**IMPORTANCE:**

Fungal growth and development are closely regulated by a variety of transcription factors. This study identified and characterized a hyphae-specific Cys_2_His_2_ zinc finger transcription factor in two *Aspergillus* species. HscA contains a Cys_2_His_2_ zinc finger domain and plays a crucial role in appropriate fungal development in *A. nidulans* and *A. flavus*. Particularly, HscA is involved in stress tolerance in both hyphal and conidial stages. We further demonstrated that HscA acts as a positive regulator of sterigmatocystin production in *A. nidulans* and is essential for proper aflatoxin B1 production in *A. flavus*. Additionally, our findings indicate that HscA is crucial for conidial formation in kernel assays, implying that HscA may function as a virulence factor. Overall, these findings enhance our understanding of mycotoxin production and fungal pathogenicity in *Aspergillus* species.

## INTRODUCTION

*Aspergillus* spp. are ubiquitous filamentous fungi found in various climates worldwide (1). Some *Aspergillus* species can cause allergies, asthma, bronchiectasis, tuberculosis, and can even spread to the brain, kidneys, liver, or other organs (2, 3). *Aspergillus*-derived diseases particularly affect immunodeficient individuals and exhibit a high mortality rate, ranging from 40% to 90% (4). Several *Aspergillus* species produce harmful mycotoxins, including aflatoxins, sterigmatocystin, and ochratoxins, which, either directly or through contaminated food, can cause various diseases in humans and animals (5, 6). Therefore, understanding the life cycle of *Aspergillus* and the mechanisms of mycotoxin production is essential to reduce their detrimental effects on human health.

*Aspergillus nidulans* is a representative model organism for studies in genetics, fungal developmental biology, and secondary metabolism (7). The spores of *A. nidulans* typically exist in a dormant state in the air and begin to grow isotropically under favorable conditions. Through a series of cell cycles, polarized spores form germ tubes, elongate, and grow vegetatively (8). The hyphal cells remain interconnected in an undifferentiated state until they encounter environmental signals such as light, temperature, carbon, and nitrogen sources, as well as various genes and proteins (9, 10). When the fungal hyphae become capable of responding to these inducing signals, they undergo asexual or sexual development and form asexual (conidiophores) or sexual structures (cleistothecia) (11, 12).

Asexual development (conidiation) is the primary reproductive pathway in *A. nidulans* (13). The mechanisms of conidiation are initiated by various genetic regulators, including the Flb proteins, Nsd proteins, and Velvet proteins (13). Proper expression of *brlA* mediates the asexual developmental switch from vegetative growth and facilitates the formation of stalks and vesicles by regulating early developmental genes (9). Influenced by BrlA, AbaA facilitates the formation of metulae and phialides during the middle stage of development and coordinates the expression levels of genes such as *wetA* and *vosA* (14, 15). In the late stage of conidiation, WetA controls the proper formation of conidiophores and the maturation of conidia (16). These regulators of asexual reproduction also perform similar roles in the toxin-producing fungus *Aspergillus flavus* (17).

Transcription factors regulate chromatin structure and gene expression by recognizing specific DNA sequences (18). The zinc cluster family is the largest group of transcription factors that regulate fungal growth, morphology, and differentiation in *Aspergillus* (19). This family includes classical Gal4-like Zn(II)_2_Cys_6_ and Cys_2_His_2_ zinc finger types, as well as non-classical types such as RING-type, PHD-type, and LIM-type (20, 21). Among these, C_2_H_2_-type transcription factors are the second-largest group of zinc cluster proteins and exhibit pleiotropic functions in *A. nidulans* biology (22). For example, RocA is a C_2_H_2_ zinc finger transcription factor that negatively regulates asexual development by repressing the transcription of *brlA* and positively regulates sexual development by activating the expression of *nsdC* in *A. nidulans* (23). VadH, which contains four C_2_H_2_ zinc finger domains, is involved in the balance between asexual and sexual development and also contributes to osmotic stress tolerance in *A. nidulans* (24). Another C_2_H_2_ zinc finger protein, CrzA, plays essential roles in fungal growth, conidiation, and resistance to ion and osmotic stress in *A. nidulans* (25).

In a previous study, we analyzed conidia-specific and hyphae-specific genes of conidia-specific transcription factors in three *Aspergillus* species (22). In this study, we identified several zinc finger transcription factors among the hyphae-specific transcription factors and generated four deletion mutants that had not been previously studied in *A. nidulans*. Among these, we further investigated the roles of a C_2_H_2_-containing transcription factor, designated HscA (hyphae-specific Cys_2_His_2_ zinc finger A).

## RESULTS

### Analysis of hyphae-specific zinc finger transcription factors in *A. nidulans*

**Fig 1:**
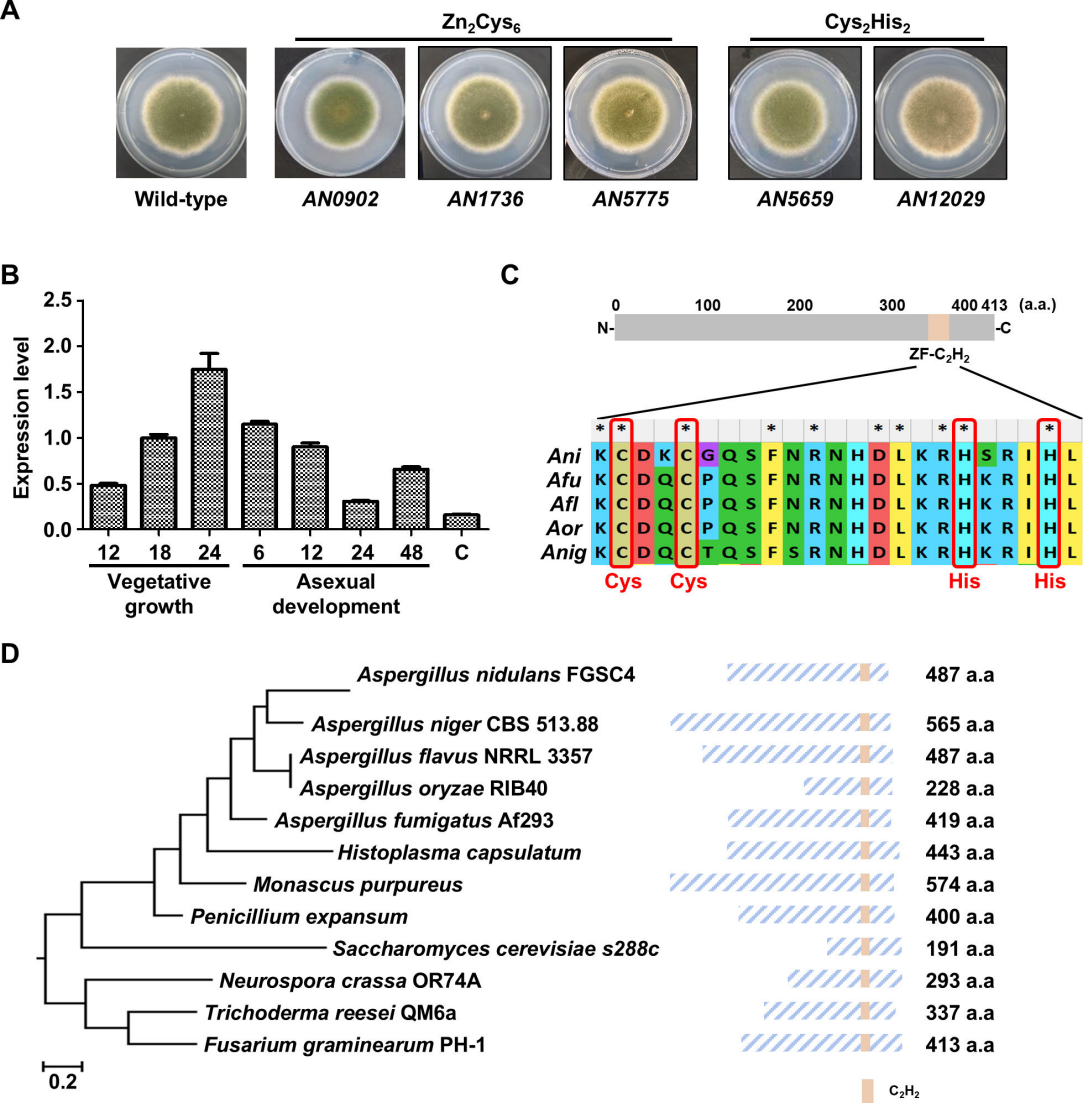
Identification of HscA in *A. nidulans.* (**A**) Colony morphology of wild-type (WT) and deletion mutant strains point-inoculated on solid MM and incubated at 37°C for 5 days. (**B**) mRNA expression levels of *hscA* during the life cycle of *A. nidulans* using qRT-PCR. Time (h) indicates the duration of incubation in liquid culture or post-asexual developmental induction. The letter “C” indicates conidia. (**C**) Domain analysis of HscA. The lower panel shows the alignment of the C_2_H_2_ zinc-finger domain from *A. nidulans* HscA and its homologs from other *Aspergillus* species (*Ani: Aspergillus nidulans*, *Afu: Aspergillus fumigatus*, *Afl: Aspergillus flavus*, *Aor: Aspergillus oryzae*, *Anig: Aspergillus niger*). (**D**) Phylogenetic tree of HscA homologs identified in fungal species including *A. niger* CBS 513.88 (XP_001395971.2), *A. flavus* NRRL 3357 (XP_041140193.1), *A. oryzae* RIB40 (XP_001816961.3), *A. fumigatus* Af293 (XP_747886.1), *Histoplasma capsulatum* (QSS50878.1), *Monascus purpureus* (TQB77170.1), *Penicillium expansum* (XP_016593862.1), *Saccharomyces cerevisiae* s288c (NP_010539.1), *Neurospora crassa* OR74A (XP_959452.3), *Trichoderma reesei* QM6a (XP_006966625.1), and *Fusarium graminearum* PH-1 (XP_011320037.1). The phylogenetic tree was generated using MEGA X software based on the alignment data from ClustalW algorism and the maximum likelihood method with the Poisson correction model. The bootstrap consensus tree inferred from 1,000 replicates represents the evolutionary history of the taxa analyzed. Right panels present domains of HscA homologs in various fungal species.

Previous transcriptomic studies have identified conidia- or hyphae-specific genes in *A. nidulans*, *A. fumigatus*, and *A. flavus* (22). In this study, we analyzed hyphae-specific transcription factors across the three *Aspergillus* species and identified a total of 13 hyphae-specific zinc finger transcription factors: six GAL4-like Zn(II)_2_Cys_6_ transcription factors and seven Cys_2_His_2_ transcription factors (Table 1). Among them, five hyphae-specific zinc finger transcription factors had not been previously characterized. To investigate the roles of these five unstudied hyphae-specific zinc finger proteins, we constructed deletion mutants and examined their colony phenotypes in *A. nidulans*. As shown in Fig. 1A, the fungal colony of the Δ*AN12029* mutant exhibited abnormal pigmentation compared to the wild-type strain. Based on these observations, we further analyzed the functions of *AN12029* in *A. nidulans*.

**TABLE 1:** List of zinc finger transcription factors significantly upregulated in the hyphae (H) compared to conidia (C) across three *Aspergillus* species (22)

TF family (*n*)	*A. nidulans*			*A. fumigatus*		*A. flavus*	
	Gene	Name	log_2_FC(C/H)	Gene	log_2_FC(C/H)	Gene	log_2_FC(C/H)
Zn_2_Cys_6_ (6)	*AN0902*		−4.05	*Afu1g15680*	−3.61	*AFLA_083820*	−1.37
	*AN1736*		−3.87	*Afu2g00880*	−4.3	*AFLA_104780*	−2.81
	*AN1848*	*nosA*	−2.92	*Afu6g07010*	−2.93	*AFLA_025720*	−6.76
	*AN3075*	*oefC*	−4.35	*Afu3g09670*	−3.51	*AFLA_085170*	−1.18
	*AN5170*	*rosA*	−1.08	*Afu6g07010*	−2.93	*AFLA_025720*	−6.76
	*AN5775*		−3.85	*Afu6g06535*	−2.78	*AFLA_037760*	−8.00
Cys_2_His_2_ (7)	*AN1652*	*msnA*	−3.18	*Afu4g09080*	−4.27	*AFLA_110650*	−5.67
	*AN2421*	*flbC*	−2.63	*Afu2g13770*	−3.81	*AFLA_137320*	−1.63
	*AN4263*	*nsdC*	−2.31	*Afu7g03910*	−1.35	*AFLA_131330*	−1.34
	*AN5583*	*aslA*	−3.69	*Afu4g11480*	−6.76	*AFLA_027460*	−1.91
	*AN5659*		−8.65	*Afu4g13600*	−2.43	*AFLA_052490*	−6.93
	*AN9492*	*amdХ*	−1.22	*Afu2g17220*	−2.44	*AFLA_002290*	−1.87
	*AN12029*		−1.86	*Afu6g01910*	−2.96	*AFLA_049760*	−1.02

### Identification of HscA

The *AN12029* gene encodes a protein that contains a hyphae-specific Cys_2_His_2_ zinc finger domain; therefore, we referred to it as HscA. To evaluate the specific expression of *hscA* in hyphae, we investigated its expression levels of *hscA* in the life cycle of *A. nidulans* using a quantitative reverse-transcription PCR (qRT-PCR) analysis. The *hscA* mRNA levels were high during vegetative growth and decreased after the onset of asexual development and in conidia (Fig. 1B). HscA consists of a single C_2_H_2_ zinc finger domain located at the C-terminus, similar to its homologs in other *Aspergillus* species (Fig. 1C). HscA is conserved in fungal species and contains a C_2_H_2_ zinc finger domain in the N-terminal region (Fig. 1D).

### The role of *hscA* in fungal development in *A. nidulans*

**Fig 2:**
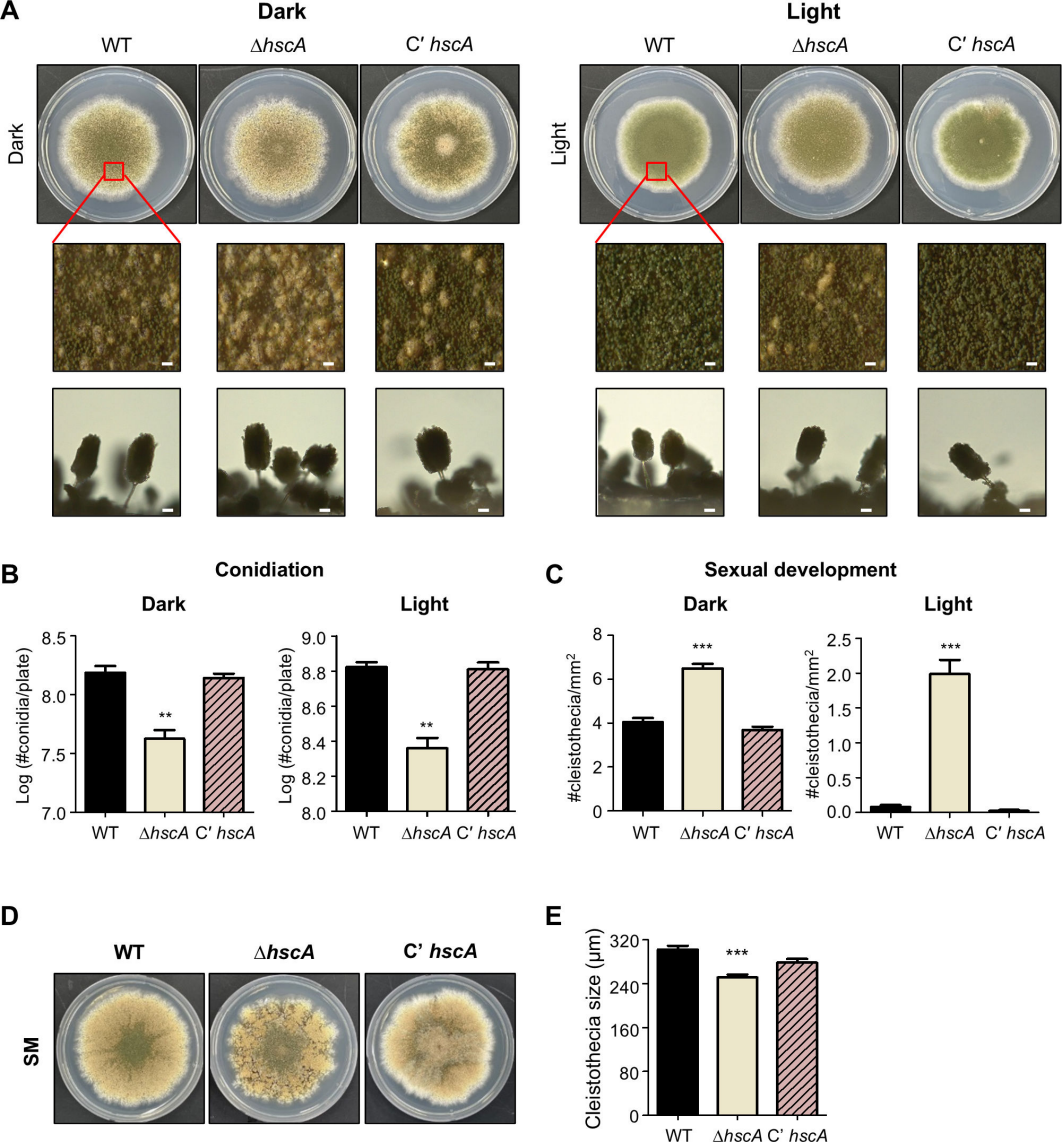
Phenotypic analysis of the Δ*hscA* mutant strains in *A. nidulans.* (**A**) Colony morphology of wild-type (TNJ36), Δ*hscA* (TYE49.1), and C’ *hscA* (TYE56.1) strains point-inoculated on solid MM and incubated for 5 days under dark or light conditions. Bottom panels show close-up views of the colony center (bar = 0.25 µm). (**B**) Quantitative analysis of the number of asexual spores shown in panel **A** (**P ≤ 0.01). (**C**) Quantitative analysis of the number of cleistothecia shown in panel **A** (***P ≤ 0.001). (**D**) Colony morphology of wild-type (TNJ36), Δ*hscA* (TYE49.1), and C’ *hscA* (TYE56.1) strains point-inoculated on solid sexual media and incubated for 7 days under dark conditions. (**E**) Measurement of cleistothecia size in wild-type, Δ*hscA*, and C’ *hscA* strains shown in panel **D** (***P ≤ 0.001).

To further investigate the roles of *hscA* in *A. nidulans*, we generated an *hscA*-complemented strain (C' *hscA*). Wild-type, Δ*hscA*, and C' *hscA* strains were point-inoculated onto solid minimal media containing 1% glucose (MMG) and incubated under dark or light conditions for 5 days. As shown in Fig. 2A, deletion of *hscA* did not affect fungal growth under either dark or light conditions. However, asexual spore production was significantly reduced in the Δ*hscA* mutant strain compared to the wild-type and C' *hscA* strains under both dark and light conditions (Fig. 2B). Additionally, the number of cleistothecia increased under both conditions (Fig. 2C). To further assess the role of HscA in sexual development, each strain was point-inoculated onto sexual media and incubated under dark conditions for 7 days (Fig. 2D). The cleistothecia produced by the Δ*hscA* strain were smaller in size compared to those formed by the wild-type and C' *hscA* strains (Fig. 2E). Overall, these results indicate that HscA is essential for appropriate fungal development in *A. nidulans*.

### The role of HscA in cell wall integrity and ion depletion stresses in *A. nidulans*

**Fig 3:**
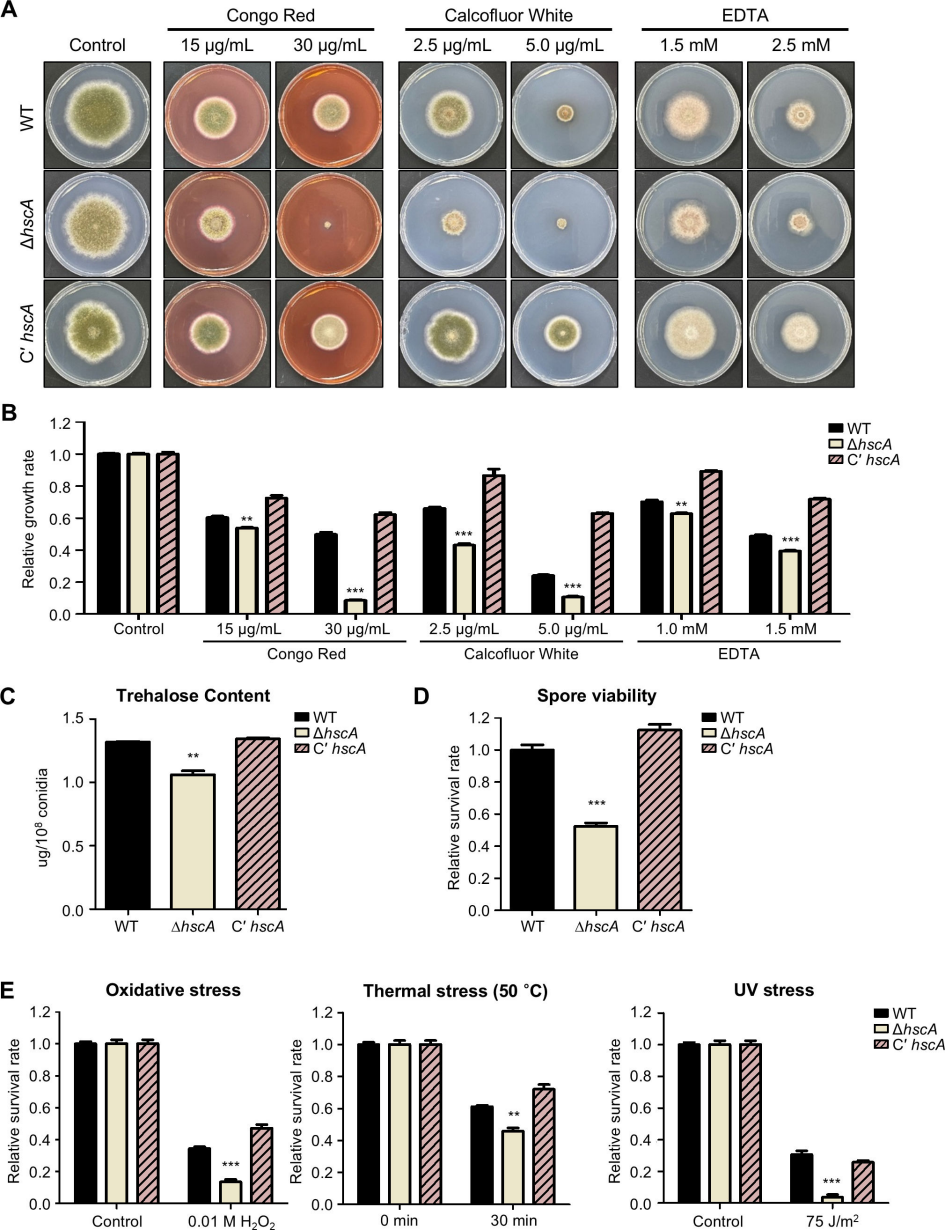
Function of HscA in stress responses in *A. nidulans.* (**A**) Colonies of wild-type (WT; TNJ36), Δ*hscA* (TYE49.1), and C’ *hscA* (TYE56.1) strains exposed to cell wall integrity and ion depletion stresses. Each strain was point-inoculated on solid MM containing various stress reagents, including Congo Red, Calcofluor White, and EDTA, and incubated at 37°C for 5 days. (**B**) Relative growth rates of the indicated strains under stress conditions. The relative growth rate was calculated based on colony diameter and normalized to that of colonies grown without stress agents (**P ≤ 0.01 and ***P ≤ 0.001). (**C**) Trehalose content in conidia of WT, Δ*hscA*, and C’ *hscA* strains (**P ≤ 0.01). (**D**) Conidial viability of WT, Δ*hscA*, and C’ *hscA* strains (***P ≤ 0.001). (**E**) Oxidative, thermal, and UV stress tolerance of wild-type, Δ*hscA*, and C’ *hscA* mutant conidia (**P ≤ 0.01 and ***P ≤ 0.001).

To investigate the roles of HscA in hyphal stress responses, we examined the tolerance of wild-type, Δ*hscA*, and C' *hscA* strains to various stress conditions. Approximately 10^5^ conidia of each strain were point-inoculated on minimal media supplemented with agents causing cell wall integrity stress (Congo Red and Calcofluor White), ion depletion stress (EDTA), osmotic stress (NaCl and KCl), oxidative stress (H_2_O_2_), and cell membrane integrity stress (SDS). The plates were incubated at 37°C for 5 days. The *hscA* deletion strains exhibited increased sensitivity against Congo Red, Calcofluor White, and EDTA (Fig. 3A). Deletion of *hscA* resulted in decreased colony diameters under these conditions (Fig. 3B). In contrast, no significant differences were observed between wild-type, C' *hscA*, and Δ*hscA* strains in response to NaCl, KCl, H_2_O_2_, and SDS (Fig. S1). These results suggest that HscA plays a crucial role in stress responses related to cell wall integrity and ion depletion in hyphae.

To further investigate the roles of HscA in conidial stress tolerance, we measured the amount of conidial trehalose, which plays a key role in stress tolerance in fungi (26). The Δ*hscA* conidia contained a lower amount of trehalose compared to wild-type and C' *hscA* conidia (Fig. 3C). Conidial viability of the Δ*hscA* conidia was also decreased compared with that of the wild-type and C' *hscA* conidia (Fig. 3D). Furthermore, the Δ*hscA* conidia were more sensitive to oxidative, thermal, and UV stresses compared to wild-type and C' *hscA* conidia (Fig. 3E). These results demonstrate that HscA plays a crucial role in stress responses in both hyphae and conidia in *A. nidulans*.

### The function of *hscA* in sterigmatocystin production in *A. nidulans*

**Fig 4:**
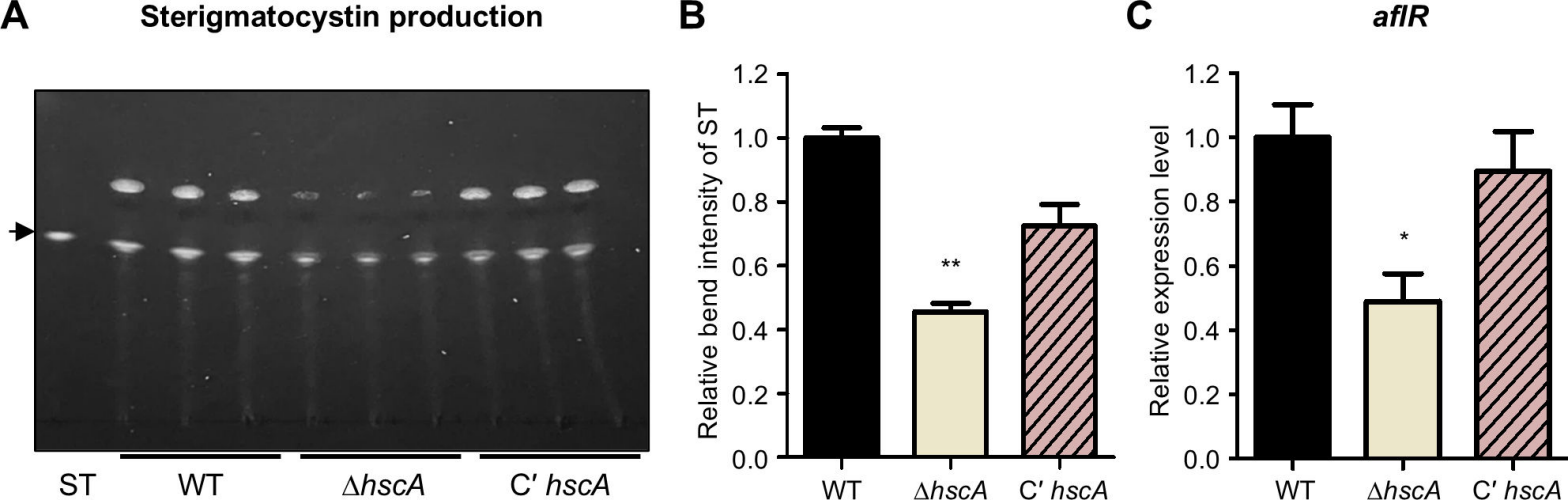
Effect of HscA on sterigmatocystin production in *A. nidulans.* (**A**) Thin-layer chromatography (TLC) analysis of sterigmatocystin in wild-type (TNJ36), Δ*hscA* (TYE49.1), and C’ *hscA* (TYE56.1) strains. The black arrow indicates the sterigmatocystin (ST) band. (**B**) Relative intensity of sterigmatocystin bands shown in panel **A** (**P ≤ 0.01). The relative band intensity of sterigmatocystin was quantified using ImageJ software. (**C**) mRNA expression levels of *aflR* in the indicated strains (*P ≤ 0.05).

To determine whether *hscA* influences sterigmatocystin production in *A. nidulans*, we extracted secondary metabolites from wild-type, Δ*hscA*, and C' *hscA* strains. Each sample was spotted onto TLC plates with sterigmatocystin as a standard. Deletion of the *hscA* strain produced less sterigmatocystin compared to the wild-type and C' *hscA* strains (Fig. 4A and B). Additionally, the mRNA level of *aflR*, the primary regulator of sterigmatocystin biosynthesis, was downregulated in the *hscA* null mutant (Fig. 4C). Furthermore, in the *hscA*-overexpressing strain (OE*hscA*), sterigmatocystin production was higher than in the control under inducing conditions (Fig. S2). These results indicate that HscA functions as an activator of sterigmatocystin production in *A. nidulans*.

### Effect of deletion of *AflhscA* on fungal development in *A. flavus*

**Fig 5:**
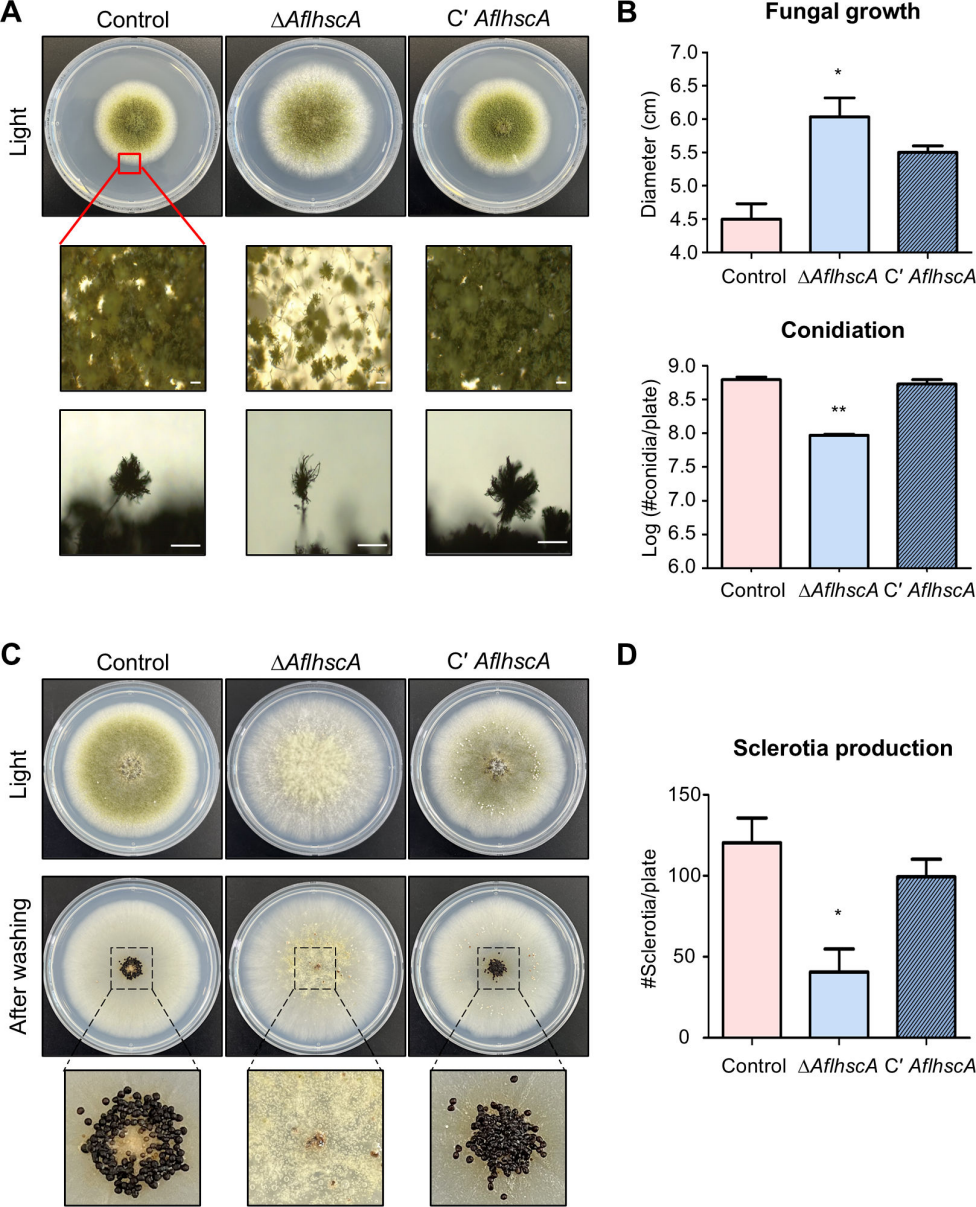
Phenotypic analysis of the Δ*AflhscA* mutant strains. (**A**) Colony morphology of control (TTJ6.1), Δ*AflhscA* (TKH1.1), and C′ *AflhscA* (TKH2.1) strains point-inoculated on solid MMYE and incubated at 37°C for 5 days under light conditions. Middle panels show close-up views of the central area of each colony (scale bars = 250 µm). Bottom panels display conidiophore images of control, Δ*AflhscA*, and C′ *AflhscA* strains. (**B**) Under light conditions, colony diameter was measured to assess fungal growth, and the total number of conidia per plate was quantified to evaluate asexual development. Error bars represent standard deviation. Statistical differences between control and mutant strains were estimated using Student’s *t*-test (*P < 0.05; **P < 0.01). (**C**) Control, Δ*AflhscA*, and C′ *AflhscA* strains were point-inoculated on solid MMYE media and incubated at 37°C for 7 days under dark conditions. After inoculation, plates were washed with 100% ethanol, and sclerotia were counted. (**D**) Quantitative analysis of sclerotia as an indicator of sexual development. All experiments were conducted in at least triplicate. Error bars indicate standard deviation. Statistical differences between control and deletion mutant strains were estimated using Student’s *t*-test (*P < 0.05).

In *A. nidulans*, HscA is required for proper asexual and sexual development. To examine the role of *A. flavus hscA* (*AflhscA*) in growth and development, the control, *AflhscA* deletion mutant (Δ*AflhscA*), and *AflhscA* complemented strains (C' *AflhscA*) were point-inoculated onto solid MMYE medium. As shown in Fig. 5A, the Δ*AflhscA* strains exhibited larger colonies than the control and C' *AflhscA* strains, and the conidiophores of the Δ*AflhscA* strains were significantly smaller than those of the control and C' *AflhscA* strains. Moreover, the deletion of *AflhscA* led to a reduction in the number of conidiospores (Fig. 5B), implying that *Afl*HscA is required for appropriate colony growth and asexual development. We then examined the function of *AflhscA* in sexual development. After incubating control, Δ*AflhscA*, and C' *AflhscA* strains onto sexual media, the number of sclerotia which act as sexual structures in *A. flavus* was counted. The Δ*AflhscA* strains produced significantly fewer sclerotia compared to the control and C' *AflhscA* strains (Fig. 5C and D). Collectively, these results indicate that *Afl*HscA is essential for normal sexual development in *A. flavus*.

### The role of *Afl*HscA in stress tolerance

**Fig 6:**
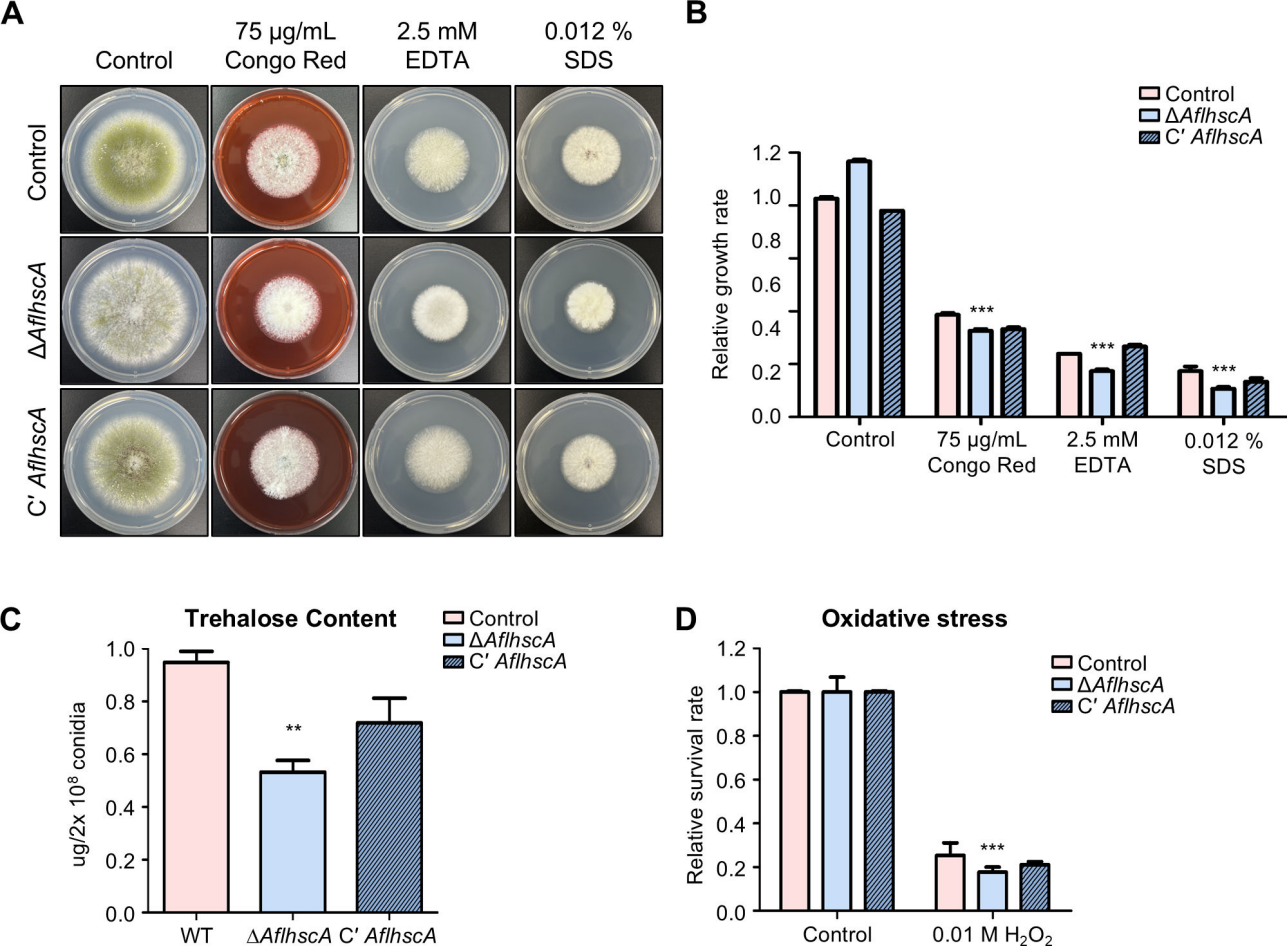
Phenotypes of the Δ*AflhscA* mutant under various stress conditions in *A. flavus.* (**A**) Colonies of control, Δ*AflhscA*, and C′ *AflhscA* strains subjected to cell wall integrity and ion depletion stresses. Each strain was point-inoculated on solid MM containing various stress reagents, including Congo Red, EDTA, and SDS, and incubated at 37°C for 5 days. (**B**) Relative growth rates of the designated strains under stress conditions. The relative growth rate was calculated by measuring the diameter of the fungal colonies and normalized to the diameter of fungal colonies grown without stress agents (***P ≤ 0.001). (**C**) Trehalose content in the conidia of control, Δ*AflhscA*, and C′ *AflhscA* strains (**P ≤ 0.01). (**D**) Oxidative stress tolerance of control, Δ*AflhscA*, and C′ *AflhscA* mutant conidia (***P ≤ 0.001).

Given that deletion of *hscA* affects stress tolerance in *A. nidulans*, we examined whether *Afl*HscA functions in a similar manner. Control, Δ*AflhscA*, and C' *AflhscA* strains were point-inoculated on solid MMYE media containing various stressors including Congo Red, EDTA, SDS, and KCl. The plates were incubated at 37°C for 5 days (Fig. 6A). The Δ*AflhscA* strains showed increased sensitivity to Congo Red, EDTA, and SDS (Fig. 6B), but not to osmotic stressors (Fig. S2). We further examined the amount of trehalose in conidia and stress resistance. We found that deletion of *AflhscA* resulted in reduced trehalose content in conidia (Fig. 6C) and increased sensitivity to oxidative stress (Fig. 6D). Overall, these findings suggest that *Afl*HscA plays an important role in stress tolerance in both hyphae and conidia of *A. flavus*.

### The function of *Afl*HscA in aflatoxin B1 production

**Fig 7:**
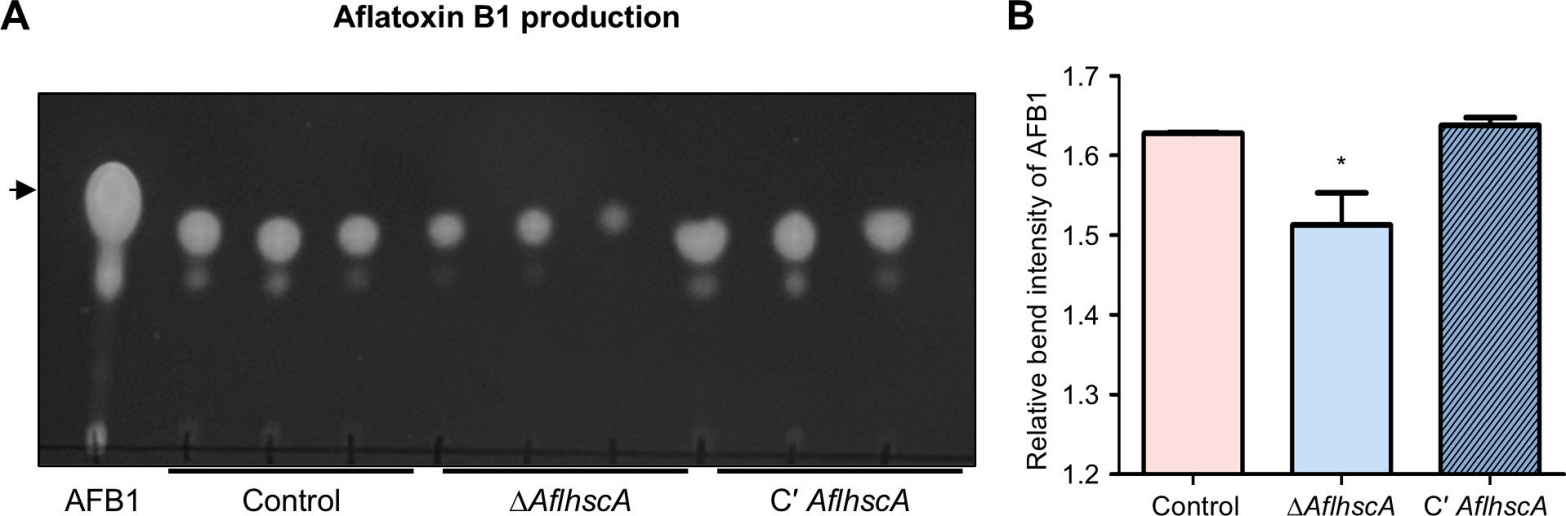
Effect of *Afl*HscA on aflatoxin B1 production in *A. flavus.* (**A**) Thin-layer chromatography (TLC) analysis of aflatoxin B1 in control, Δ*AflhscA*, and C′ *AflhscA* strains. The black arrow indicates aflatoxin B1 (AFB1). (**B**) Relative intensity of the aflatoxin B1 bands shown in panel **A** (*P ≤ 0.05). The relative band intensity of aflatoxin B1 was quantified using ImageJ software.

To investigate whether *Afl*HscA influences aflatoxin B1 production, control, Δ*AflhscA*, and C' *AflhscA* strains were inoculated into liquid CM medium and incubated at 30°C for 7 days under dark conditions. Following this, aflatoxin B1 was extracted from each sample, and the amount of aflatoxin B1 was analyzed using thin-layer chromatography. We found that the Δ*AflhscA* mutant strain produced a lesser amount of aflatoxin B1 compared to the control or complemented strains (Fig. 7). These results suggest that *Afl*HscA is crucial for proper aflatoxin B1 production.

### The role of *Afl*HscA in pathogenicity

**Fig 8:**
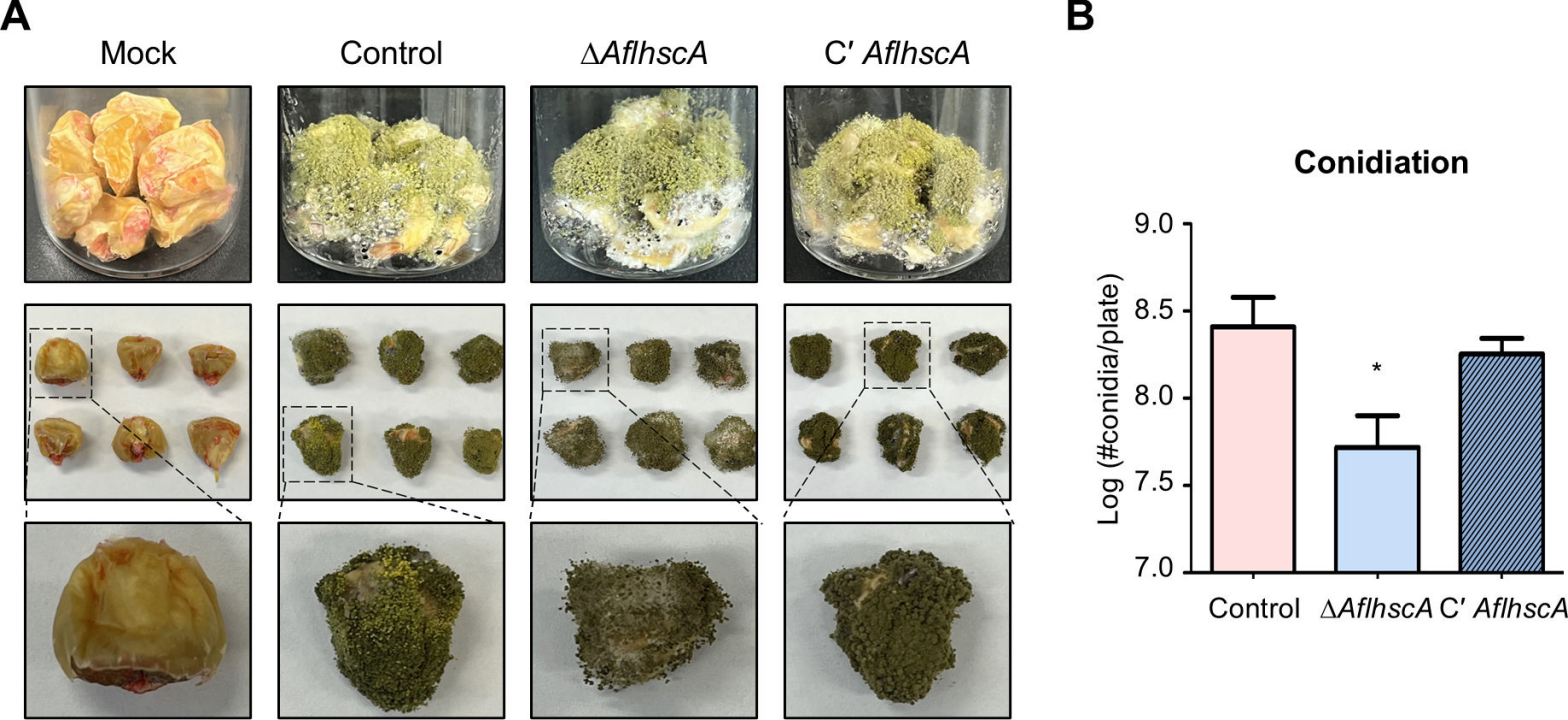
Maize kernel infection assay. (**A**) Phenotypes of uninfected corn seeds (Mock) and corn seeds infected by control, Δ*AflhscA*, and C′ *AflhscA* strains after incubation at 30°C for 7 days under dark conditions. The bottom row shows enlarged views of each maize seed. (**B**) Quantitative analysis of conidia production from each infected maize kernel. This experiment was performed in triplicate for each strain. Statistical differences between control and deletion mutant strains were assessed using Student’s *t*-test (*P < 0.05).

To investigate the role of *Afl*HscA in fungal pathogenicity, a kernel infection assay was performed. Conidia of the control, Δ*AflhscA*, and C' *AflhscA* strains were infected onto maize kernels and cultured at 30°C for 7 days under dark conditions (Fig. 8A). After incubation, the Δ*AflhscA* strain produced fewer conidia compared to the control and C’ *AflhscA* strains (Fig. 8B). However, in the Δ*AflhscA* mutant, aflatoxin B1 production was lower than in the control strain, but the difference was not statistically significant (data not shown). Overall, these results suggest that *Afl*HscA is required for proper fungal development and contributes to plant pathogenesis.

## DISCUSSION

Transcription factors have been reported to play various roles in fungal growth, development, secondary metabolite production, and pathogenicity (19, 21). In a previous study, we analyzed mRNA expression in hyphae and conidia and performed RNA-seq analysis to identify hyphae- or conidia-specific genes (22). We also previously investigated the function of the SscA transcription factor in three *Aspergillus* species (22, 27, 28). In this study, we focused on genes with higher mRNA expression in hyphae compared to conidia and investigated genes encoding proteins belonging to the zinc finger family (Table 1). Among the 13 transcription factors identified, the functions of seven (NosA, OefC, RosA, MsnA, FlbC, NsdC, AmdX, and AslA) have been previously studied (29, 30). Interestingly, these transcription factors which exhibit higher mRNA expression in hyphae than in conidia are involved in various aspects of fungal biology. For instance, MsnA is known to participate in stress responses and developmental regulation in hyphae (31). FlbC is a key transcription factor that regulates the expression of *brlA*, a transcription factor that initiates asexual development (32). Additionally, NosA, RosA, and NsdC have been implicated in sexual reproduction (33–35). AslA promotes normal asexual development and represses sterigmatocystin biosynthesis in *A. nidulans* (36). Furthermore, AslA is also associated with osmotic stress resistance and vacuolar transporters (37). In this study, we further examined five transcription factors whose functions remain unknown (Fig. 1A). Among them, the deletion mutant of *AN12029* showed significant phenotypic differences, leading us to designate this gene as *hscA*. We subsequently conducted a detailed investigation of *hscA* in *A. nidulans* and *A. flavus*.

In this study, we investigated the function of *hscA* in the growth and development of *A. nidulans* and *A. flavus*. In *A. nidulans*, the *hscA* deletion mutant did not affect growth but resulted in a reduction in conidia production, an increase in the number of sexual reproductive structures, and a decrease in cleistothecia size (Fig. 2). In *A. flavus*, the *hscA* deletion mutant exhibited increased colony growth but showed abnormal conidiophore structures, along with a decrease in the production of both conidia and sclerotia (Fig. 5). These findings suggest that HscA plays a crucial role in the production of both asexual and sexual reproductive structures, although its specific role may differ between *Aspergillus* species.

HscA was also found to play a similar role in stress responses in both *A. flavus* and *A. nidulans* (Fig. 3 and 6). The *hscA* deletion mutants of both species exhibited reduced growth on media containing cell wall inhibitors or EDTA, indicating their involvement in stress sensitivity. Furthermore, the *hscA* deletion mutants exhibited decreased trehalose contents and increased sensitivity to oxidative stress in the conidia of both *Aspergillus* species. These findings suggest that HscA plays a crucial role in stress responses in both hyphae and asexual spores and that this function appears to be well conserved across *Aspergillus* species.

*Aspergillus* species produce various mycotoxins, including sterigmatocystin, aflatoxins, and ochratoxins (5). Notably, sterigmatocystin produced by *A. nidulans* and aflatoxin B1 produced by *A. flavus* share similar chemical structures and biosynthetic pathways (38). In this study, we found that *hscA* deletion reduced sterigmatocystin production in *A. nidulans* (Fig. 4). Similarly, the deletion of *hscA* in *A. flavus* led to decreased aflatoxin B1 production (Fig. 7), suggesting that HscA may play a similar role in the biosynthesis of both sterigmatocystin and aflatoxin B1. However, results from the kernel assay indicated that aflatoxin B1 production in the *hscA* deletion mutant of *A. flavus* was comparable to that of the control strain. This suggests that under certain environmental conditions, the reduction in aflatoxin B1 production caused by *hscA* deletion may be compensated. Therefore, while HscA plays an important role in aflatoxin B1 biosynthesis, it is not essential for its production.

In summary, we investigated hyphae-specific zinc finger transcription factors and characterized a hyphae-specific C_2_H_2_-type zinc finger transcription factor, HscA, in the model organism *A. nidulans* and the toxigenic fungus *A. flavus*. Our phenotypic analyses demonstrate that HscA is essential for regulating fungal differentiation, tolerating environmental stresses, and producing mycotoxins. Further studies will provide deeper insights into the genetic regulatory mechanisms of HscA in *A. nidulans*, and additional research will help clarify its conserved roles in other *Aspergillus* species.

## MATERIALS AND METHODS

### Strains, media, and culture conditions

Fungal strains used in this study are listed in Table 2. For general purposes, *A. nidulans* strains were cultured on liquid or solid minimal media with 1% glucose (MMG) at 37°C. To induce sexual development, *A. nidulans* strains were grown on solid sexual media at 37°C (39). Each *A. flavus* strain was cultured on MMG supplemented with 0.1% yeast extract (MMYE) at 37°C. For auxotrophic strains, uridine/uracil or pyridoxine was added to the medium as required. *Escherichia coli* DH5α cells were grown in Luria-Bertani (LB) medium supplemented with ampicillin (100 µg/mL) for plasmid amplification.

**TABLE 2:** *Aspergillus* strains used in this study

Strain name	Relevant genotype	Reference
*A. nidulans*		
FGSC4	Wild type, *veA*^+^	FGSC^*a*^
RJMP1.59	*pyrG89*; *pyroA4*, *veA*^+^	(40)
TNJ36	*pyrG89*; *AfupyrG* ^+^; *pyroA4*, *veA*^+^	(32)
THS30	*pyrG89*; *AfupyrG* ^+^	(41)
TYE1.1~3	*pyrG89*; *pyroA4*; *∆AN0902*::*AfupyrG*^+^, *veA*^+^	This study
TYE46.1~3	*pyrG89*; *pyroA4*; *∆AN5775*::*AfupyrG*^+^, *veA*^+^	This study
TYE47.1~3	*pyrG89*; *pyroA4*; *∆AN5659*::*AfupyrG*^+^, *veA*^+^	This study
TYE48.1~3	*pyrG89*; *pyroA4*; *∆AN1736*::*AfupyrG*^+^, *veA*^+^	This study
TYE49.1~3	*pyrG89*; *pyroA4*; *∆AN12029*::*AfupyrG*^+^, *veA*^+^	This study
TYE56.1~2	*pyrG89*; *pyroA::hscA(p):: hscA::FLAG_3x_::pyroA^b^; ∆ hscA*::*AfupyrG*^+^, *veA*^+^	This study
TYE123.1	*pyrG89*; *AfupyrG*^+^; *pyroA::alcA(p):: hscA::FLAG_3x_::pyroA^b^*, *veA*^+^	This study
*A. flavus*		
NRRL 3357	Wild type (WT)	FGSC^*a*^
NRRL 3357.5	*pyrG^−^*	(42)
TTJ6.1	*pyrG*^−^; Δ*AflpyrG*::*AfupyrG^+^*	(43)
TKH1.1-2	*pyrG*^−^; Δ*AflhscA*::*AfupyrG^+^*	This study
TKH2.1-2	*pyrG*^−^; *AflhscA(p)*::*AflhscA*::*FLAG_4x_*::*ptrA;* Δ*AflhscA*::*AfupyrG^+^*	This study

^*a*^ Fungal Genetic Stock Center.

^*b*^ The 3/4 *pyroA* marker allows targeted integration at the *pyroA* locus.

### Generation of gene deletion mutants

The oligonucleotide primers used in this study are detailed in Table 3. The double-joint PCR (DJ-PCR) method was used to generate gene deletion mutant strains (44). The 5′ and 3′ flanking regions of each gene were amplified using the primer pairs 5′DF/3′ tail or 5′tail/3′ DR, respectively, with *A. nidulans* FGSC4 genomic DNA (gDNA) or *A. flavus* NRRL 3357 gDNA as templates. The *A. fumigatus pyrG* (*AfupyrG*) marker was amplified with the primer pair OHS1542/OHS1543 using *A. fumigatus* AF293 gDNA as a template. The final PCR constructs for each gene deletion were amplified with the primer pair 5′ NF/3′ NR of the corresponding gene. The deletion cassettes were introduced into *A. nidulans* RJMP1.59 or *A. flavus* NRRL 3357.5 protoplasts, which were generated using Vinoflow FCE lysing enzyme (Novozymes, Bagsvaerd, Denmark) (45, 46). Each resulting strain was independently isolated and finally verified through PCR, restriction enzyme treatment, and qRT-PCR analyses.

**TABLE 3:** Oligonucleotides used in this study

Name	Sequence (5′ → 3′)^*a*^	Purpose
OHS1542	CCTGGTCTTTGGTTTGGTACACC	5′ *AfupyrG* marker_F
OHS1543	CGACTGGCAGGAGATGATCC	3′ *AfupyrG* marker_R
OHS0214	GAGGCATGGCATTGGCTTTG	5′ *AN0902* DF
OHS0216	*GGCTTTGGCCTGTATCATGACTTCA* CGAGGTTGAGAACTCGAGCCTTC	3′ *AN0902* with *AfupyrG* tail
OHS0217	*TTTGGTGACGACAATACCTCCCGAC* CGACGTGGAACGTTTAATTGGC	5′ *AN0902* with *AfupyrG* tail
OHS0215	TGGTTGCGGTGGTTGAGGAAG	3′ *AN0902* DR
OHS0218	GAGCTATAACCCTTGTCGATGGC	5′ *AN0902* NF
OHS0219	GAGGAAGGAGTGTGCGGTGTC	3′ *AN0902* NR
OHS0441	GGGCATCCCAGTGCTACTTT	5′ *AN0902* RT_F
OHS0442	TTCTCCGCAGCAACCCTATC	3′ *AN0902* RT_R
OHS1614	GGC CTA TGT GGT CCT CGA	5′ *AN1736* DF
OHS1574	GCTAACCCTCTTACCGCAGT	3′ *AN1736* with *AfupyrG* tail
OHS1575	*GGCTTTGGCCTGTATCATGACTTCA* GGTTCAAGCGTGTCGAACTAG	5′ *AN1736* with *AfupyrG* tail
OHS1576	*TTTGGTGACGACAATACCTCCCGAC* GTGCTGTTTACCAGGCGAG	3′ *AN1736* DR
OHS1577	GATGATGCGGTTTCGACCG	5′ *AN1736* NF
OHS1578	CACTATCTCGCAACGAACGG	3′ *AN1736* NR
OHS1579	GCGAACCCTAATGCCAGG	5′ *AN1736* RT_F
OHS1580	CTGGTGGAGAACCAGCCATA	3′ *AN1736* RT_R
OHS1581	GTTGGTGAGCTCTGGTGAAC	5′ *AN5775* DF
OHS1582	GAGAGACCAGGCCTCGAG	3′ *AN5775* with *AfupyrG* tail
OHS1583	*GGCTTTGGCCTGTATCATGACTTCA* GAGTCGCGAGCTGGTCT	5′ *AN5775* with *AfupyrG* tail
OHS1584	*TTTGGTGACGACAATACCTCCCGAC* GAGTCGCGAGCTGGTCT	3′ *AN5775* DR
OHS1585	GTATTCTGCGACGCCGTG	5′ *AN5775* NF
OHS1586	ATCTCTGAAGCGAGCTCCT	3′ *AN5775* NR
OHS1587	GGACTATCGACTGGAGGTCC	5′ *AN5775* RT_F
OHS1588	CGAGCCCACTATCCGAGTAT	3′ *AN5775* RT_R
OHS1589	GGCTGGGATAGACCTGCTTT	5′ *AN5659* DF
OHS1590	GGATCAGGCTCGTGGTCT	3′ *AN5659* with *AfupyrG* tail
OHS1591	*GGCTTTGGCCTGTATCATGACTTCA* GTGCACAGCTGTACCAACC	5′ *AN5659* with *AfupyrG* tail
OHS1592	*TTTGGTGACGACAATACCTCCCGAC* GTGCACAGCTGTACCAACC	3′ *AN5659* DR
OHS1593	GCGAAGTAGTAGAGAGCTGCG	5′ *AN5659* NF
OHS1594	GACCTTCCAGGTAGGTCCAG	3′ *AN5659* NR
OHS1595	CAGTGCAGGAACCAATACAAGC	5′ *AN5659* RT_F
OHS1596	CGTCGAAGATACTGCCAGGT	3′ *AN5659* RT_R
OHS1597	TGACGAGATGGAACCGGAAT	5′ *AN12029* DF
OHS1598	CATGGACAGTCCGCTGTC	3′ *AN12029* with *AfupyrG* tail
OHS1599	*GGCTTTGGCCTGTATCATGACTTCA* CAAGGGTCGCCAGGTTTG	5′ *AN12029* with *AfupyrG* tail
OHS1600	*TTTGGTGACGACAATACCTCCCGAC* CAAGGGTCGCCAGGTTTG	3′ *AN12029* DR
OHS1601	CCTGCTCTTCCTGCAAGTG	5′ *AN12029* NF
OHS1602	GTATCTGACCGCTCGCTG	3′ *AN12029* NR
OHS1603	CATCGAAGTGCCACCAGTC	5′ *AN12029* RT_F
OHS1604	CACCGGCTCATATAGTGGGA	3′ *AN12029* RT_R
OHS1812	AATT **GAATTC** CAAGATCATGGACAGTCCGC	5′ *hscA* with promoter and *EcoR1*
OHS1813	AATT **GAATTC** CTCGCCGCTCTCAACCTT	3′ *hscA* with *EcoR1*
OHS2748	AATT **GCGGCCGC** ATGGGGACGGGCCTCG	5′ *hscA* with *Not1*
OHS1805	AATT **GCGGCCGC** CTCGCCGCTCTCAACCTT	5′ *hscA* with *Not1*
OHS0044	GTAAGGATCTGTACGGCAAC	5′ *actin* RT_F
OHS0045	AGATCCACATCTGTTGGAAG	3′ *actin* RT_R
OHS0580	CAAGGCATGCATCAGTACCC	5′ *brlA* RT_F
OHS0581	AGACATCGAACTCGGGACTC	3′ *brlA* RT_R
OHS0779	ATTGACTGGGAAGCGAAGGA	5′ *abaA* RT_F
OHS0780	CTGGGCAGTTGAACGATCTG	3′ *abaA* RT_R
OHS0599	GCGCGAAGAAGACTTCAAC	5′ *aflR* RT_F
OHS0600	TGCAATAACTGCCGACGAC	3′ *aflR* RT_R
OHS3274	CCTGTCAACCACGTTGTCGG	5′ *AflhscA* DF
OHS3275	*GGCTTTGGCCTGTATCATGACTTCA* GAACCGCAAGAGCAGCCA	3′ *AflhscA* with *AfupyrG* tail_R
OHS3276	*TTTGGTGACGACAATACCTCCCGAC* CCCTTGATCTCGAGCGAC	5′ *AflhscA* with *AfupyrG* tail_F
OHS3277	CTTCTGGTCCATCCTGGC	3′ *AflhscA* DR
OHS3278	TCCCGTTCCATTTGCTCG	5′ *AflhscA* nested NF
OHS3279	TCCAACAAGCCGACGCAG	3′ *AflhscA* nested NR
OHS3280	CGATCCGACCTCGTCCTAC	5′ *AflhscA* RT_F
OHS3281	GTCGCCGAATTTCGATGGAG	3′ *AflhscA* RT_R
OHS3318	AATT **GCGGCCGC** CTCCGTACTTACTCTCGC	5′ *AflhscA* with promoter and *Not*I
OHS3319	AATT **GCGGCCGC** GCCATGGTCCAGGTCTTT	3′ *AflhscA Not*I

^*a*^ Tail sequences are shown in italics. Restriction enzyme sites are in bold.

### Construction of *hscA*-complemented strains

To generate the Δ*hscA* complemented strain in *A. nidulans*, the wild-type *hscA* gene region, including its predicted promoter, was amplified using the primer pair OHS1812/OHS1813, digested with *EcoR*I, and cloned into pHS13 (47). The resulting plasmid, pYE13.1 was then introduced into the recipient Δ*hscA* strain TYE49.1, yielding TYE56.1~2. The complemented strains were screened by PCR and confirmed by quantitative reverse transcription- PCR (qRT-PCR).

To generate *AflhscA*-complemented strains in *A. flavus*, the promoter and open reading frame (ORF) region of *AflhscA* was amplified using primers OHS3318 and OHS3319. The PCR product and pYES1 (27) were digested with *Not*I and cloned. The resulting plasmid, pKH1.1, was transformed into the Δ*AflhscA* mutant, yielding strains TYE56.1~2. Ultimately, strain TKH2.1-2 was obtained as a complemented strain and verified through PCR, qRT-PCR, and phenotypic analyses.

### Nucleic acid isolation and qRT-PCR analysis

To isolate gDNA, approximately 10^6^ conidia of each strain were inoculated into 2 mL of liquid MMG supplemented with 0.5% yeast extract and incubated at 37°C for 24 h. The mycelial mat was harvested, and squeeze-dried, and genomic DNA was extracted as previously described (46).

For total RNA preparation, fresh conidia were inoculated into liquid MM or MMYE and incubated at 37°C for 12, 18, and 24 h. To induce developmental stages, mycelia grown for 18 h were harvested, washed, and transferred to solid MM or MMYE, and incubated at 37°C under either light or dark conditions. Samples were collected at designated time points and stored at −80°C until RNA extraction (48).

For total RNA extraction, each sample was homogenized using a Mini-Bead Beater (BioSpec Products Inc., Bartlesville, OK, USA) with 1 mL of TRIzol reagent (Geneall, Seoul, South Korea) and 0.3 mL of glass beads (Daihan Scientific, Wonju, South Korea). After centrifugation, the supernatant was transferred to a new tube and mixed with an equal volume of cold isopropanol. Following a second centrifugation and removal of the supernatant, the RNA pellet was washed with 70% ethanol treated with diethyl pyrocarbonate (DEPC, Bioneer, Daejeon, South Korea). cDNA was synthesized from total RNA using reverse transcriptase (Promega, Madison, WI, USA). qRT-PCR assay was performed using the iTaq Universal SYBR Green Supermix (Bio-Rad, Hercules, CA, USA) on a CFX96 Touch Real-Time PCR system (Bio-Rad, USA). The β-actin gene was used as a control.

### Stress sensitivity assay

For *A. nidulans*, approximately 10^5^ conidia of each strain were point-inoculated onto solid MM supplemented with various stress-inducing agents and incubated at 37°C for 5 days. The following stress agents were used: cell wall integrity stress: 15 and 30  µg/mL Congo Red (Sigma, St Louis, MO, USA), 2.5 and 5.0 µg/mL Calcofluor White (Thermo Fisher, Waltham, MA, USA); ion depletion stress: 1.5 and 3.0 mM ethylenediaminetetraacetic acid (EDTA; Bioneer, Daejeon, South Korea). Relative growth rates were calculated using the following formula: relative growth rate = fungal colony diameter with stress agent/fungal colony diameter without stress agent. Each experiment was performed in triplicate per strain.

For *A. flavus*, each strain was point-inoculated onto solid MMYE medium containing 75 µg/mL Congo Red (Sigma, USA), 2.5 mM EDTA (Bioneer, South Korea), and 0.012% sodium dodecyl sulfate (SDS), and incubated at 37°C under dark conditions for 5 days. After incubation, colony sizes (colony diameter, cm) were measured. All experiments were performed in triplicate.

### Sterigmatocystin extraction and thin-layer chromatography (TLC) analysis

For sterigmatocystin extraction from *A. nidulans*, approximately 10^5^ conidia of each strain were inoculated into 5 mL of liquid complete medium (CM) and cultured at 30°C for 7 days. After incubation, 5 mL of CHCl_3_ (Duksan, Seoul, Republic of Korea) was added to each culture, and the samples were centrifuged to separate the organic and aqueous phases. The organic phase was transferred to clean glass vials and evaporated in an oven for 24 h. The dried residue was resuspended in 50 µL of CHCl_3_ and spotted onto a TLC silica gel plate with a fluorescence indicator (Kieselgel 60, 0.25 mm; Merck, Rahway, NJ, USA). The TLC plate was resolved in toluene:ethyl acetate:acetic acid (8:1:1, vol/vol) and treated with 1% aluminum hydroxide hydrate (Sigma, St Louis, MO, USA). The developed plates were exposed to ultraviolet illumination at 366 nm and photographed. Spot intensity of sterigmatocystin was quantified using ImageJ software. Experiments were performed in triplicate for each strain.

### Aflatoxin B1 extraction and TLC analysis

For aflatoxins B1 extraction from *A. flavus*, approximately 2 × 10^6^ of each strain were inoculated into 5 mL of liquid CM and incubated at 30℃ under dark conditions for 7 days. After incubation, 5 mL of chloroform (Duksan, Ansan, South Korea) was added, and the samples were vigorously mixed using a Voltex mixer. The mixture was centrifuged to separate the aqueous phase and organic phase. The organic phase was collected, filtered through filter paper, and mixed with distilled water (ddH_2_O). Following vortexing and centrifugation, the lower layer was collected and treated with Na_2_SO_4_, and 2 mL of chloroform was added, and the mixture was vortexed and centrifuged again. The final organic phase was evaporated overnight in an oven. Chloroform (100 µL) was added to dissolve the dried samples, and the prepared samples were loaded onto a TLC silica gel plate (Kieselgel 60, 0.25 mm; Merck KGaA, Darmstadt, Germany). The TLC plate was placed in a chamber containing a chloroform: acetone (9:1, vol/vol) solvent. Aflatoxin B1 bands were visualized under UV light at 366 nm.

### Kernel assay

To assess the fungal pathogenicity of each strain, two-day-old conidia were harvested using ddH_2_O containing 0.02% Triton X-100 (Sigma, USA), and diluted to a concentration of 2 × 10^6^ conidia/mL. Kernels were washed with 70% ethanol for 5 min using a RotoBot Mini Programmable Rotator (Benchmark Scientific, Sayreville, NJ, USA), followed by treatment with 6% sodium hypochlorite (Samchun, Pyeongtaek, Republic of Korea) for 10 min. The kernels were then rinsed with ddH_2_O for 5 min; this process was repeated five times. After drying, each maize kernel was infected with conidia from each strain. Inoculated kernels were incubated at 30℃ incubator under light conditions for 7 days. To quantify conidia production from infected kernels, a hemocytometer was used.

### Microscopy

Colony photographs were captured using a Pentax MX-1 digital camera. Photomicrographs were captured using a Leica DM500 microscope equipped with a Leica ICC50 E camera and Leica Application Suite X software.

### Statistical analysis

Statistical differences between wild-type or control strains and mutant strains were evaluated using Student’s unpaired *t*-test. Data are presented as mean ± standard deviation (SD). *P* values < 0.05 were considered statistically significant (*P ≤ 0.05; **P ≤ 0.01; ***P ≤ 0.001).

## SUPPLEMENTAL MATERIAL

Supplemental figures msphere.00254-25-s0001.pdf Fig. S1 and S2.

ASM does not own the copyrights to Supplemental Material that may be linked to, or accessed through, an article. The authors have granted ASM a non-exclusive, world-wide license to publish the Supplemental Material files. Please contact the corresponding author directly for reuse.
