# Supplementary material for: The hyphae-specific C2H2 transcription factor HscA regulates development, stress response, and mycotoxin production in Aspergillus species
Source: mSphere. 2025 Jun 10;10(7):e00254-25. doi: 10.1128/msphere.00254-25 (PMC12306176; doi:10.1128/msphere.00254-25)
Supplement: Supplemental figures — Fig. S1 and S2. [file msphere.00254-25-s0001.pdf]

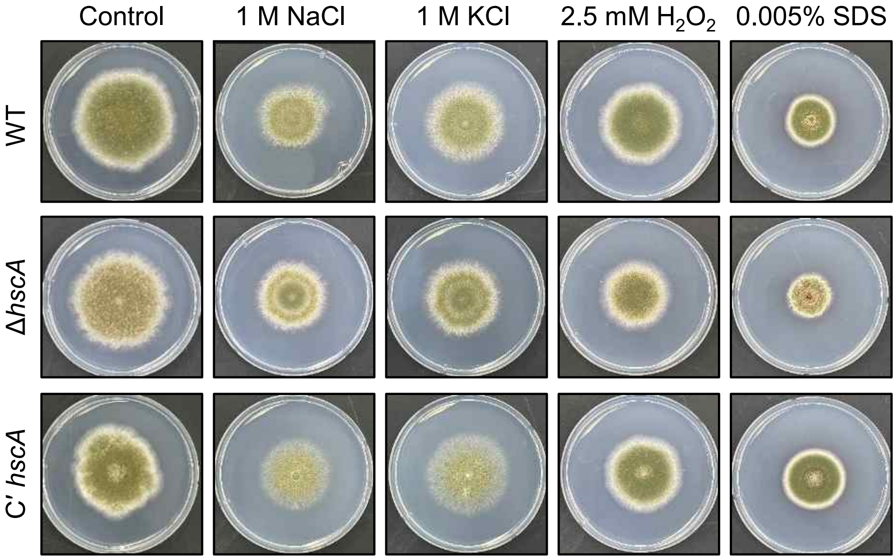

Figure S1. Phenotypes of the Δ*hscA* mutant in various stress conditions. Colonies of WT (TNJ36), Δ*hscA* (TYE49.1), and C' *hscA* (TYE56.1) strains subjected to osmotic and SDS stresses.

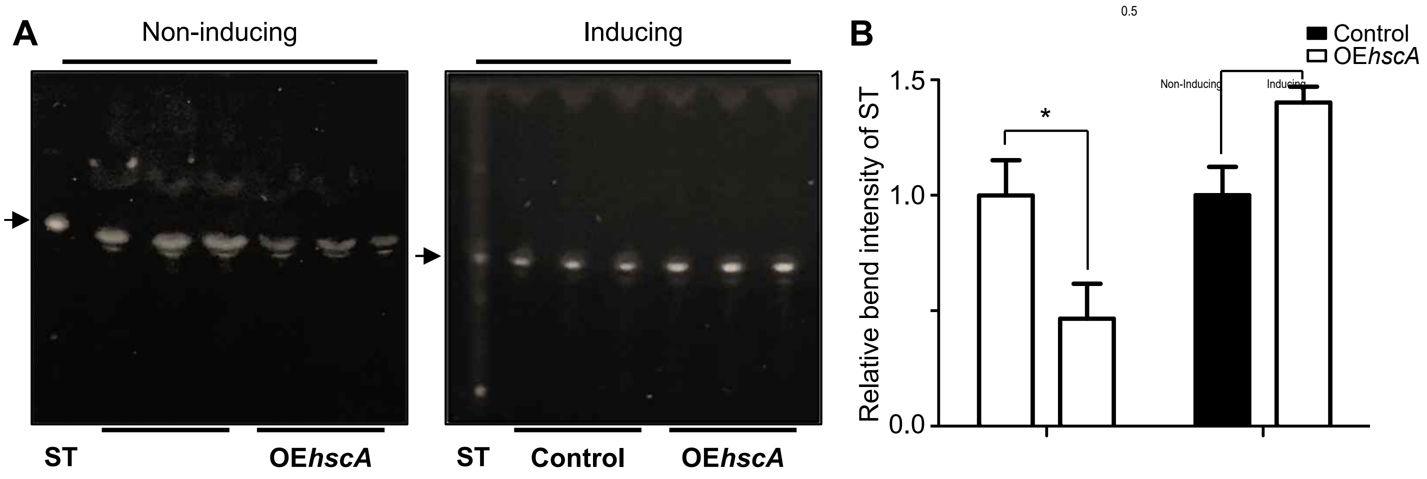

Figure S2. Effect of HscA on sterigmatocystin production. (A) TLC analysis of sterigmatocystin in control (THS30) and OE*hscA* (TYE123.1) strains under non-inducing or inducing condition. (B) Relative intensity of the ST bands shown in (A). Spot intensity of sterigmatocystin was quantified using ImageJ software (* $p \leq 0.05$, ** $p \leq 0.01$).
